# Effect of Hydroxyapatite and Bovine Serum Albumin on the Antibacterial Activity of MTA

**Published:** 2011-11-15

**Authors:** Zahed Mohammadi, Sousan Shalavi

**Affiliations:** 1. Department of Endodontics, Dental School, Hamedan University of Medical Sciences, Hamedan, Iran.; 2. Iranian Centre for Endodontic Research, Shahid Beheshti University of Medical Sciences, Tehran, Iran.

**Keywords:** Bovine Serum Albumin, Hydroxyapatite, Inactivation, MTA

## Abstract

**INTRODUCTION:**

The purpose of this study was to compare the inhibitory effect of bovine serum albumin (BSA) and hydroxyapatite (HA) on the antibacterial activity of white-colored MTA (WMTA) against Staphylococcus (S.) aureus and Streptococcus (S.) mutans after 24 and 72 hours.

**MATERIALS AND METHODS:**

All materials were prepared according to the manufacturer’s directions immediately before testing. The antibacterial effect of each group (WMTA, WMTA+BSA and WMTA+HA) was determined by measuring the diameter of zone of inhibition in millimeters after incubation at 37°C for 24 and 72 hours in a humid atmosphere. Each test was repeated three times. Data were analyzed using ANOVA and Tukey’s test.

**RESULTS:**

In the 24 hours samples as well as in 72 hours samples, the antibacterial activity of MTA+HA group was significantly greater than two other groups against S. aureus (P<0.05). However, the antibacterial activity of MTA+HA group against S. mutans was not significantly different from the MTA group in 24 hours as well as 72 hours samples. BSA reduced the antibacterial activity of MTA against both tested bacteria in the 24 and 72 hour samples (P<0.05).

**CONCLUSION:**

The products studied exhibited antibacterial activity. However, in both time intervals, the MTA+HA group exerted the greatest activity against S. mutans and S. aureus.

## INTRODUCTION

An optimal root-end filling material should give impervious apical sealing and be resistant to desorption, radiopaque and biocompatible to the periapical tissues [1]. Currently, MTA is the most favorable root-end filling material. It consists of 50-75% (wt) calcium oxide and 15-25% silicon dioxide. These two components together comprise 70-95% of the cement. When these raw materials are blended they produce tricalcium silicate, dicalcium silicate, tricalcium aluminate, and tetracalcium aluminoferrite [2]. On addition of water the cement hydrates to form silicate hydrate gel [2]. It has been shown that on hydration, MTA produces calcium hydroxide. Thus, it can be concluded that both MTA and calcium hydroxide may have similar mechanism of action [3]. Studies have demonstrated the inhibitory effect of dentin powder, bovine serum albumin (BSA), and hydroxyapatite (HA) on the antibacterial activity of calcium hydroxide [4][5]. On the other hand, one study showed that dentin enhanced the antibacterial activity of MTA [6]. However, there is no study on the effect of BSA and HA on the antibacterial activity of MTA. Therefore, the purpose of this study was to assess the effect of BSA and HA on the antibacterial activity of MTA using agar diffusion test (ADT).

## MATERIALS AND METHODS

The materials used in the present study were white-colored MTA (Dentsply, Tulsa Dental, Tulsa, OK, USA), HA (Bio-Rad Laboratories, Richmond, CA, USA), and BSA (Sigma Chemical Co., St. Louis, MO, USA).MTA was prepared at concentration of 50mg/mL by dilution with distilled water. Both inhibitors (BSA and HA) were used in powder form. A total of 28mg powder was suspended in 50µL water. 50µL of the different inhibitor suspensions were thoroughly mixed and incubated with 50µL MTA in sealed test tubes at 37°C for 1 hour before adding 50µL of the bacterial suspension, giving a total volume of 150µL. One control group consisted of 50µL sterile water instead of BSA or HA and the other control group consisted of 50µL sterile water instead of MTA. The suspensions were carefully mixed and incubated at 37°C in air.

The microorganisms used in this study included Staphylococcus (S.) aureus and Streptococcus (S.) mutans. Overnight cultures of the microorganisms were used. The bacteria were grown in Tryptic Soy Broth and were adjusted to the turbidity of a 0.5Mc Farland BaSo_4_ standard (≈1.5×10^8^ colony forming unit (CFU) mL^-1^). Thirty petri dishes containing Trypticsoy agar enriched with 5% defibrinated sheep blood and supplemented with hemin and vitamin K were seeded with bacteria. Seeding was done using sterile cotton-tipped applicators that were brushed across the agar surface. Three wells of 5mm depth and 6mm diameter were punched in each agar plate and filled with freshly mixed materials. Fifteen wells were used for each group. The plates were then maintained at room temperature for two hours for pre-diffusion of the material.

Later, the antimicrobial effect of each material was determined by measuring the diameter of the inhibition zone in millimeters after incubation at 37°C for 24 hours in a humid atmosphere. Each test was repeated three times. Data were analyzed using ANOVA and Tukey’s test. Differences at the 5% level were considered statistically significant.

## RESULTS

All materials tested demonstrated some antibacterial activity. HA increased the efficacy of MTA against both bacterial species (P<0.05). However, BSA decreased the efficacy of MTA on both tested bacterial species (P<0.05). The mean diameters of microbial inhibition for each sealer against each microorganism are shown in Table 1 and Table 2.

**Table 1:** Means diameters of bacterial growth inhibition zones (mm) in 24 hours samples

**Bacterial Species**	**N**	**WMTA**	**WMTA+BSA**	**WMTA+HA**
		Mean±SD	Mean±SD	Mean±SD
* S. aureus *	13	25±3.81	15.72±2.82	29.83±2.67
* S. mutans *	13	35.20±3.29	24.65±3.66	34.75±3.63

**Table 2:** Means diameters of bacterial growth inhibition zones (mm) in 72 hours samples

**Bacterial Species**	**N**	**WMTA**	**WMTA+BSA**	**WMTA+HA**
		Mean±SD	Mean±SD	Mean±SD
* S. aureus *	13	17±1.29	13.20±2.57	21.67±2.75
* S. mutans *	13	22.50±2.95	21.80±1.89	21.46±3.42

## DISCUSSION

Microorganisms are considered to be the primary etiologic agents in endodontic diseases [7][8]. The agar diffusion test has been widely used to evaluate the antibacterial activity of dental materials [9][10][11]. Several factors that are relevant for the diffusion capacity of materials in agar must be considered, such as the contact between the experimental material and agar, molecular weight, size and shape of the antimicrobial agent, load and concentration of test material, agar gel viscosity, and ionic concentration in relation to the medium. Furthermore, control and standardization of inoculation density, evaluation of results, selection of agar medium, selection of micro-organisms, depth of agar medium, incubation temperature of plates, and reading point of inhibition haloes are also restricting factors affecting the dynamics and variability of diffusion tests in an agar medium [12]. Nevertheless, if most of these variables are carefully controlled, then consistent and reproducible results may be obtained [9]. However, clinical inferences should be drawn with strict caution when based on in vitro findings only.

Although aerobic and facultative bacteria are usually minor constituents of primary infections, they have been found in cases in which the treatment had been protracted, in flare-ups, and associated with endodontic failures. Therefore it is important to evaluate the antimicrobial activity of endodontic materials against aerobic and facultative bacteria in addition to anaerobic bacteria. Furthermore, test microorganisms used in the present study were utilized in several studies to evaluate the antimicrobial effects of root canal sealers [12][13][14].

It has been shown that on hydration, MTA produces Ca(OH)_2_. Thus, it can be concluded that both MTA and Ca(OH)_2_ may have similar mechanism of action [2][3]. The antimicrobial activity of Ca(OH)_2_ is related to the release of hydroxyl ions in an aqueous environment [2]. Hydroxyl ions are highly oxidant free radicals that show extreme reactivity with several biomolecules [15]. This reactivity is high and indiscriminate, so this free radical rarely diffuses away from sites of generation [15].

In the present study, the antibacterial activity of MTA and the possible inhibitory effect of BSA and HA on its antibacterial activity was assessed after 24 hours and 72 hours. Findings showed that HA enhanced the antibacterial activity of MTA significantly, whereas BSA reduced it. It should be noted that the present study was the first study on the effect of HA and BSA on the antibacterial activity of MTA. Haapasalo et al. found that dentin powder abolished the antibacterial activity of calcium hydroxide against Enterococcus (E.) faecalis completely [4]. In another study, Portenier et al. found that besides dentin powder, HA and BSA inactivated the antibacterial property of calcium hydroxide as well [5].

Zhang et al. found that dentin powder enhanced the antibacterial activity of MTA against E. faecalis [6]. Furthermore, Zehnder et al. found that dentin potentiated the activity of bioactive glass against E. faecalis [16].

According to Eldeniz et al. both fresh-mixed and set MTA demonstrated antibacterial activity against S. aureus, E faecalis, and Pseudomonas (P.) aeroginosa, concurring with the findings of the present study [16]. Al-Hezaimi et al. revealed that the antibacterial activity of grey-colored MTA against E. faecalis was significantly greater than white-colored MTA [17]. Sipert et al. showed that MTA and Portland cement inhibited the growth of several microorganisms including Candida (C.) albicans, S. aureus, E. faecalis, Staphylococcus epidermidis, and P. aeroginosa, which is in accordance to our study [18]. Ribeiro et al. demonstrated that antibacterial activity of MTA against E. coli under aerobic conditions promoted by induction of reactive oxygen species [19]. In another study, Ribeiro et al. found that E. faecalis was susceptible to Gray MTA as well as calcium hydroxide after incubation in an aerobic condition [20]. They proposed that oxygen-riched media favors the antimicrobial activity of MTA. Estrela et al. showed no antibacterial activity of MTA and Portland cement against S. aureus, E. faecalis, P. aeroginosa, Bacillus subtilis, and C. albicans [21]. The antibacterial effect of MTA has been attributed to its high pH or to substances that are released from MTA into the media. On the other hand, using ADT, Miyagak et al. found that MTA showed no antimicrobial activity which is in contrast to the findings of the present study [22].

It should be noted that the antibacterial activity of set materials was decreased compared to the freshly-mixed ones. This can be attributed to decrease in the releasing of antibacterial components from the tested materials.

The results of the agar diffusion test do not depend only on the toxicity of the material for the particular microorganism, but are highly influenced by the diffusing ability of the material across the medium.

## CONCLUSION

Within the limitations of the present in vitro study, BSA reduced the antibacterial activity of MTA significantly, whereas HA increased the efficacy of MTA against S. aureus and did not affect its efficacy against Streptococcus mutans significally.
